# A systematic review and meta-analysis of the effects of combined aripiprazole on glycolipid metabolism in schizophrenia

**DOI:** 10.3389/fpsyt.2024.1496986

**Published:** 2025-01-10

**Authors:** Tianbao Wei, Limei Jiang, Ruilin Zhang, Hang Su, Zhenjie Sun, Junwei Sun

**Affiliations:** ^1^ Yulin City Veterans' Hospital, Yulin, Guangxi, China; ^2^ School of Mental Health, Shanxi Medical University, Taiyuan, Shanxi, China; ^3^ Psychological Counseling Center of the Second People's Hospital of Suzhou City, Suzhou, Anhui, China; ^4^ School of Humanities and Social Sciences, Shanxi Medical University, Taiyuan, Shanxi, China; ^5^ Taiyuan Psychiatric Hospital, Taiyuan, Shanxi, China

**Keywords:** schizophrenia, aripiprazole, combination therapy, glucolipid metabolism, review

## Abstract

**Background:**

Current research on aripiprazole adjunct therapy suggests potential benefits in improving psychiatric symptoms and metabolic disorders in patients with schizophrenia. However, the evidence remains limited due to the scarcity of research and a lack of detailed analysis on glucose and lipid metabolism indicators. This study aims to systematically review and analyze randomized controlled trials (RCTs) to evaluate the effects of aripiprazole combination therapy on both psychiatric symptoms and glycolipid metabolism.

**Materials and methods:**

A systematic search of PubMed, EMBASE, and Web of Science databases was conducted to identify randomized controlled trials (RCTs) investigating the impact of aripiprazole combination therapy on glycolipid metabolism and clinical symptoms.

**Results:**

Adjuvant treatment with aripiprazole reduced blood glucose, triglycerides, total cholesterol, and LDL levels in patients with schizophrenia, but had no significant effect on HDL levels. In addition, the study results showed a significant improvement in metabolic parameters at short-term (≤ 8 weeks) and dosing doses >15 mg. However, aripiprazole adjuvant therapy may lead to worsening of clinical symptoms, so caution is required when using it clinically.

**Conclusions:**

Aripiprazole adjunct therapy shows potential benefits in improving both psychiatric symptoms and metabolic parameters, but more comprehensive research is needed to solidify these findings, particularly regarding glycolipid metabolism indicators.

## Highlights

- The combined dose of aripiprazole >15 mg for less than 8 weeks can significantly improve metabolism.
- Metabolic improvement was more pronounced in the olanzapine group with combined aripiprazole therapy.
- The metabolic parameters included in some studies are not comprehensive, resulting in insufficient information of some indicators, which may lead to inaccurate outcomes.

## 1 Introduction

Schizophrenia is a serious mental condition that causes considerable impairment in everyday functioning due to irregularities in thinking, cognition, behavior, and emotions (1). In addition, the life expectancy of people with schizophrenia is reduced by 20 years (2).

Antipsychotic medications are an effective treatment for schizophrenia, but sadly, they can cause about 30% of patients to experience weight gain and disorders of glucolipid metabolism (3, 4), as abnormalities in metabolism usually manifest themselves within the first three months of therapy. The order of antipsychotic medications related to metabolic abnormalities is as follows: Clozapine > Olanzapine > Risperidone = Quetiapine > Ziprasidone = Aripiprazole (5). The biggest factor that affects the metabolism of antipsychotic medications may be the H1 receptor (6). Of course, some other receptors cannot be ignored (7). Although the degree of negative effects of different antipsychotic medications varies, almost all antipsychotic medications can cause weight gain (8). These medications bind to G-protein receptors on the postsynaptic membrane, activate D2 receptors, 5-HT receptors, M-cholinergic receptors, and H receptors that are coupled to them, and these receptors are distributed in the hypothalamus, liver, pancreatic β-cells, adipose tissue, and skeletal muscle. By affecting neuropeptides and 5′-AMP-activated protein kinase (AMPK) activity, which in turn leads to a series of metabolic abnormalities through signal transduction (9). Abnormalities in glycolipid metabolism may lead to adverse consequences (10, 11) and may reduce patients’ compliance with the medication, accompanied by cognitive function impairment (12). Therefore, it is very important to intervene in the metabolic disorders of patients with schizophrenia.

On the other hand, antipsychotic medications are not effective for all patients with schizophrenia, with at least 30% of patients not responding well to the medications. These patients show no clinical improvement after treatment with a sufficient amount and duration of a medication, and even after switching to another medication. This condition is called refractory schizophrenia, and clozapine is usually chosen as the last option for treatment (13). Nevertheless, 40–70% of those with refractory schizophrenia are unresponsive to clozapine, even though the serum concentration of clozapine has reached therapeutic levels (14).

Monotherapy with antipsychotic medications is an evidence-based treatment plan, suitable for both acute and maintenance treatment. If monotherapy fails after two adequate doses and courses, and there are no absolute contraindications, a trial of clozapine should be considered. If treatment with adequate dose and duration has not resulted in significant clinical improvement, it is necessary to rule out other possible reasons for reduced treatment effectiveness. At this point, consider using long-acting injectables or measuring serum drug concentrations to confirm whether there is poor medication adherence or if the drug has reached an effective therapeutic concentration. In the final resort, consider using combination therapy with antipsychotic medications (15). Of course, during treatment, it is also important not to overlook the option of modified electroconvulsive therapy (MECT) (16).

In clinical practice, the combination of antipsychotic medications is inevitable, as drug switching strategies may not always lead to the expected therapeutic effects. An open-label short-term study of 33 schizophrenic patients switching from other antipsychotic medications to aripiprazole showed that all patients successfully switched (17). However, another 6-month follow-up study of schizophrenic patients (n=127) switching from combined treatment to single treatment found that only two-thirds of patients successfully switched to a monotherapy regimen, while the remaining one-third had to resume combined treatment. Furthermore, there are other studies that oppose drug switching strategies (18–20). These findings suggest that for some patients, the combination of antipsychotic medications may be necessary, as monotherapy may not achieve the expected therapeutic effects (21).

It is typical for clinicians to prescribe several antipsychotic drugs at once, a practice known as antipsychotic polypharmacy (APP) (22). Studies have shown that the incidence of combined treatment is 23.1% (23). Previous research has found that combined antipsychotic medication therapy has more side effects than monotherapy (24). A study of 364 patients who received second-generation antipsychotic combination therapy and monotherapy found that combination therapy was associated with a higher prevalence of metabolic syndrome (50.0% vs. 34.3%, p=0.015) and TG/HDL (50.7% vs. 35.0%, p=0.016) (25). Most treatment recommendations discourage the use of many medicines at once because of the complications that arise as a result of taking multiple drugs for the same condition, alongside higher incidence, greater intensity of side effects, reduced adherence, higher drug interaction ricks, and erratic medications (26). In addition, combined medications might increase the total dose of medication, raise the receptor occupancy threshold, but lack additional efficacy, and might increase the incidence of side effects at high doses (27).

Several meta-analyses of RCTs on the combination therapy of antipsychotic drugs evaluated the feasibility of APP, but the results were conflicting. Some of the studies supporting combination medication were usually short-term, open-label studies, whereas some high-quality studies tend not to support the idea of combination medication. Interestingly, these meta-analyses usually reserved their opinions on the combination treatment of aripiprazole, believing that the combination treatment regimen of aripiprazole may have a facilitating effect (15, 28–35). Nevertheless, there were studies that said it was too soon to draw the conclusion that aripiprazole was more successful than other medicines in terms of weight reduction, lowering the burden of severe side events, and improving clinical outcomes, instead using a small number of experimental results to compare (34). In addition, a random controlled trial exploring the efficacy and safety of aripiprazole combination therapy for schizophrenia (n=4457) found that combination aripiprazole had a significant advantage in weight gain, but did not explore its effects on glycolipid metabolism (36).

Thus, aripiprazole has attracted the attention of scholars. The scope of this study goes beyond that of just examining a potential strategy for metabolic regulation, but also to focus on improving metabolic problems while effectively controlling psychiatric symptoms, which will be more beneficial for clinical decisions. Previous studies on aripiprazole adjunct therapy mainly focused on weight research, with limited attention to glycolipid metabolism. The evidence for aripiprazole-assisted therapy is limited, and there is no clear clinical guideline. Thus, we did a comprehensive review and meta-analysis of RCTs to synthesize the current information and give a foundation for treatment choices about the impact of combination aripiprazole on glycolipid metabolism in patients with schizophrenia.

## 2 Methods

### 2.1 Search strategy

The review protocol has already been registered in PROSPERP (PROSPERO 2023 CRD42023447923). This study was reported according to the Preferred Reporting Items for Systematic Reviews and Meta-Analyses (PRISMA) guidelines (37).

We conducted literature searches in three electronic databases, PubMed, Embase, and Web of Science(from the date of creation to 24 August 2023), with the following search strategy: (schizophrenia) AND (((Aripiprazole) AND ((((adjunctive) OR (additional)) OR (combine)) OR (coadministration))) AND ((((((((metabolic) OR (body mass index)) OR (Weight)) OR (fasting glucose)) OR (fasting triglycerides)) OR (total cholesterol)) OR (high-density lipoprotein)) OR (low-density lipoprotein)))(with no filters).

### 2.2 Study selection

Trials, specifically randomized controlled ones or RCTs, using adjunctive atypical antipsychotic medication in individuals with schizophrenia or schizoaffective disorders and indicators of glycolipid metabolism were included in this analysis. All studies did not restrict language. Primary outcomes included fasting glucose, fasting triglycerides, total cholesterol, high-density lipoprotein, and low-density lipoprotein. Psychological symptoms were assessed using the Positive and Negative Symptom Scale (PANSS), or CGI-S scale. As well as drug-related adverse effects were assessed. Studies were excluded if they used non-pharmacological treatments, if they used second-hand data, if the study procedures or results were unclear, and if the subjects had comorbidities or if the subjects were children or adolescents.

Two researchers (WTB and JLM) performed literature searches independently according to the selection criteria, screened all titles and abstracts, and after discussing eligible articles, compiled a list and obtained the full text of each article. Any subsequent exclusions were discussed case by case.

### 2.3 Quality assessment

The Cochrane bias risk assessment tool was used to assess all included studies for random sequence generation, allocation concealment, blinding of participants and personnel, incomplete outcome data, blinding of outcome assessment, selective reporting, and other potential biases (38). We rated the studies’ potential for bias as either low, high, or unclear based on the evaluation findings.

### 2.4 Data extraction

Standardized data and characteristics extracted from eligible studies, including authors of the literature, year of publication, source, sample size, intervention, primary outcome indicators (serum glucose, HDL, cholesterol, triglycerides, and LDL), secondary indicators (body mass index, body weight), results of psychiatric symptom assessment, and results of medication-related adverse effects.

### 2.5 Statistical analyses

For analyses of continuous outcomes, where positive psychiatric symptoms, negative symptoms, and total CGI-S scores were reported, values for metabolic indicators were reported as weighted mean differences (WMD) with 95% confidence interval (CI), whereas those for metabolic indicator values were reported as WMD with 95% CI. For analyses of dichotomous outcomes, summary statistics were expressed as odds ratios (OR) with 95%CI. When combining continuous results, we weighted each study using the inverse variance method. We utilized the I^2^ statistic to gauge the degree of dissimilarity across the studies, with values over 50% suggesting substantial variation. To further explore potential causes of variation, we also conducted sensitivity analyses, meta-regression with continuous variables, and subgroup analyses. For all analyses, we employed two-sided tests, with *P*<0.05 signifying statistical significance.

The meta-analyses were conducted using the free software RevMan version 5.4 from the Cochrane Collaboration database. Data from the graphs were extracted using GetData Graph Digitizer 2.2.6 (http://www.getdata-graph-digitizer.com).

## 3 Results

### 3.1 Literature search and characteristics

**Figure 1:**
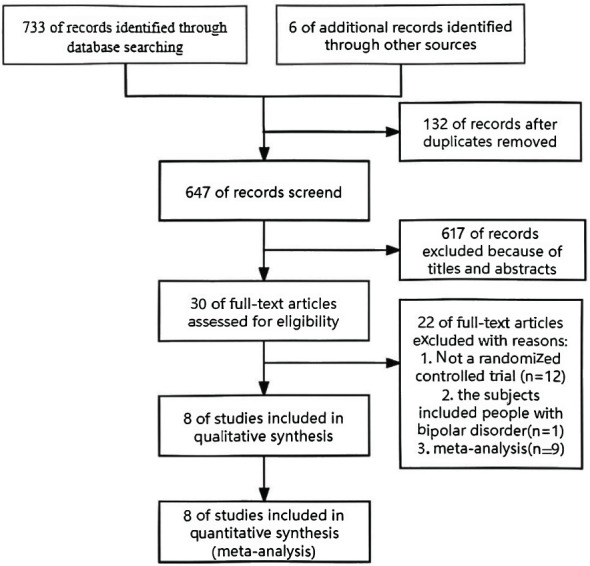
Flow chart of literature search and screening.

Seven hundred seventy-three trials were collected from the database for this meta-analysis, and an additional six publications were added after manual searches of their references. 749 trials were excluded after abstract evaluation. After reviewing the entire texts of 30 possible trials, 22 were deemed ineligible, leaving just eight publications that fulfilled the inclusion requirements. **Figure 1** provides the steps involved in the screening procedure.

The primary features of these research are summarized in **Table 1**. The eight RCT-based studies examining the use of aripiprazole as an additional medication for schizophrenia patients also looked into its impact on glycolipid metabolism. Of the eight RCTs, five studies lasted 8 weeks of treatment, one study lasted 10 weeks and two studies lasted 16 weeks. Patients were randomly allocated to receive either an active antipsychotic drug (n=2) or a placebo (n=6). Primary outcomes differed between RCTs: 8 RCTs included reported blood glucose, triglycerides and cholesterol, 7 RCTs reported HDL, and 6 RCTs reported LDL, but one randomized controlled trial reported metabolic parameters using a median that could not be extracted, and therefore metabolic parameters were excluded from this literature. Five RCTs reported PANSS total scores and 3 RCTs reported CGI total scores. Adverse reactions such as inability to sit still, back pain, gastrointestinal reactions, dizziness, salivation, dry mouth, malaise, headache, insomnia, somnolence, vomiting, restlessness and nausea-induced vomiting, and extrapyramidal reactions were reported in the included literature, but the same adverse reaction was reported by up to four studies. Regarding the loss to follow-up in various studies, among which 3 studies have 0 participants lost to follow-up (39–41), 2 studies have 1 participant lost to follow-up (41, 42), 1 study has 2 participants lost to follow-up (43), 1 study has 6 participants lost to follow-up (44), and 1 study has 8 participants lost to follow-up (45).

**Table 1:** Characteristics of the included studies.

Author	Year	country	Concurrent therapy	mean intervention time(week)	N	Trial group					Control group				Outcomes	Participants characteristics
						n1	Mean age (SD)(year)	Sex (male/ female)	Drug	Mean dose (SD)(mg/d)	n2	Mean age(year)	Sex (male/ female)	Drug		
JChang et al.	2008	Korea	clozapine	8	62	29	33.2(8.2)	22/7	Aripiprazole	15.5(7.1);	32	31.7(7.4)	26/6	placebo	BPRS, metabolic parameters, Over-arousal, Orthostatic blood pressure	Patients with DSM-IV schizophrenia who had a history of treatment failure or partial response to long-term clozapine treatment
Fan et al.	2012	USA	clozapine	8	38	16	44.3(8.2)	13/3	Aripiprazole	15	14	44.2(8.9)	9/5	placebo	metabolic parameters, Over-arousal, Drowsiness, Itching, Headache, Chest pain, Back pain, Nasal congestion, Hypersalivation, Nausea, Vomiting, Stomach discomfort	Subjects were recruited from the Freedom Trial Clinic
Jia et al.	2022	China	olanzapine	8	68	34	32~65	18/16	Aripiprazole	10+	34	29~63	19/15	Blank control	PANSS score and Clinical efficacy, metabolic parameters, Gastrointestinal reactions, Dizziness, Nausea, Sleepiness, Thirst	schizophrenic patients
Henderson et al.	2009	USA	olanzapine	10	16	15	49(8)	10/5	Aripiprazole	15	15	49(8)	10/5	placebo	Prolactin and Prolactin-Related Adverse Effects, Abdominal pain, Anorexia, Bruising easily, Constipation, Diarrhea, Dizziness, Dry mouth, Enuresis, Fever, Headache, Insomnia, Malaise, Mucosal ulceration, Nausea, Rash, Restlessness, Salivation, Sedation, Sore throat, Stiffness, Vomiting	Premenopausal Women With Psychosis
Fleischhacker et al.	2010	USA	clozapine	16	207	106	37.6(10.9)	68/40	Aripiprazole	11.1	99	40.5(9.9)	66/33	placebo	metabolic parameters, body weight and clinical efficacy, Nausea, Diarrhoea, Vomiting, Anxiety, Insomnia, Headache, Nasopharyngitis, Cough, Akathisia	schizophrenia patients
JKane et al.	2009	USA	quetiapine or risperidone	16	323	168	44.1(11.3)	99/69	Aripiprazole	10.3	155	44.4(12.0)	99/56	placebo	PANSS total score and metabolism parameter, Fatigue, Headache, Insomnia, Akathisia, Somnolence, Psychotic disorder, Back pain	chronic, stable schizophrenia or schizoaffective disorder
Chen et al.	2015	China	risperidone	8	60	30	34.50(8.91)	15/15	Aripiprazole	20	30	34.03(9.47)	15/15	placebo	PANSS score, metabolic parameters and Clinical efficacy, Dry mouth	Both outpatients and hospitalized patients from Henan Mental Hospital
Zhao et al.	2015	China	risperidone	8	113	54	28.94(7.84)	21/35	Aripiprazole	10	53	30.41(8.26)	25/32	Blank control	metabolic parameters, Clinical efficacy, Extrapyramidal events, Akathisia, Dry mouth, Nausea, Somnolence, Drooling	Both outpatients and hospitalized patients from Henan Mental Hospital Overweigh and Obese risperidone-treated Schizophrenia Patients

**Figure 2:**
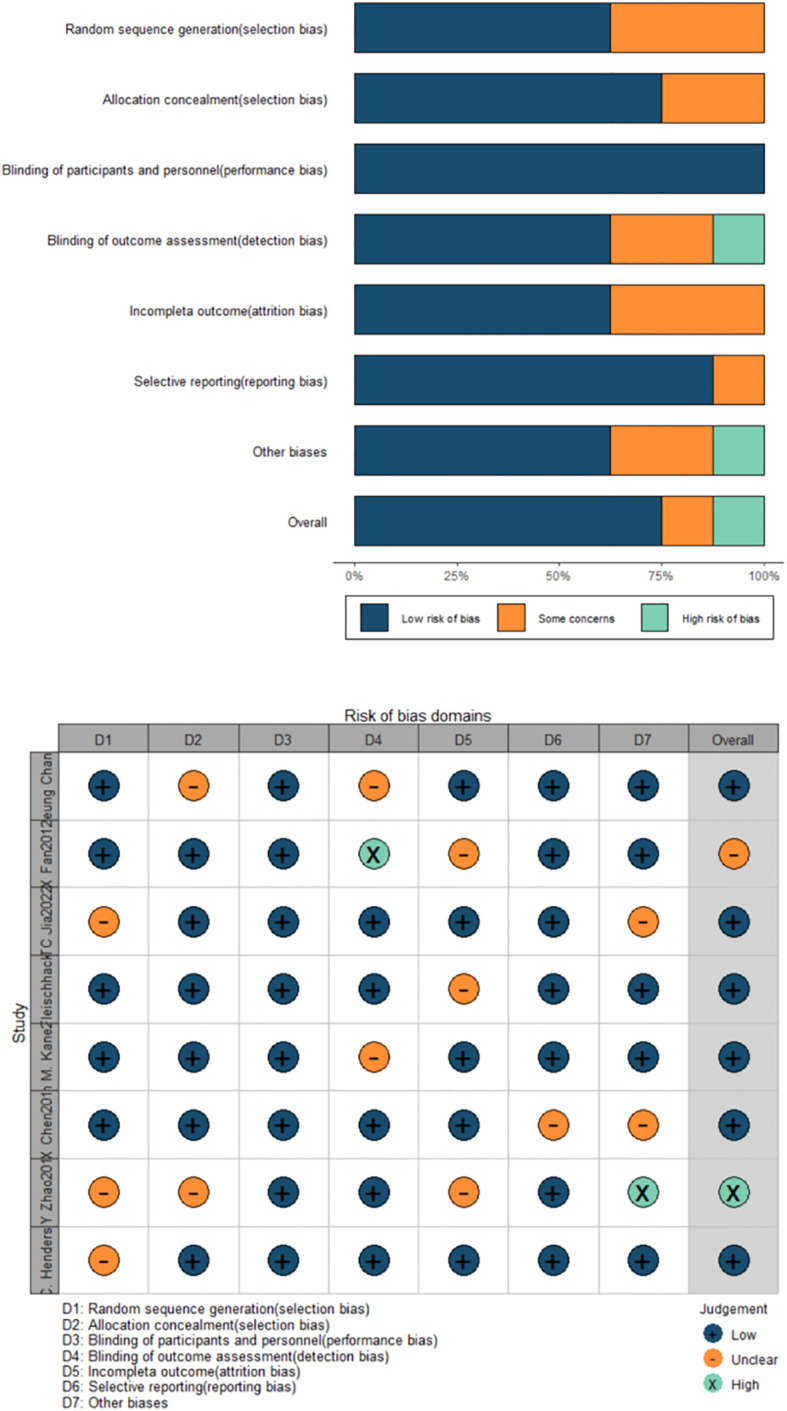
Results of the quality assessment of the studies included in the systematic review.

The Cochrane bias risk assessment result (**Figure 2**) showed that of the total 8 studies, 5 were classified as low risk of randomization, 3 were unclear; 6 RCTs clearly described allocation concealment, but 2 were unclear; all studies used blinded methods; 3 RCTs had unclear bias risks in incomplete outcome data. Eventually, 6 RCTs were judged as low risk, 1 as high risk, and 1 was unclear. Among them, 5 studies were supported by pharmaceutical companies.

### 3.2 Effect on blood glucose levels

**Figure 3:**
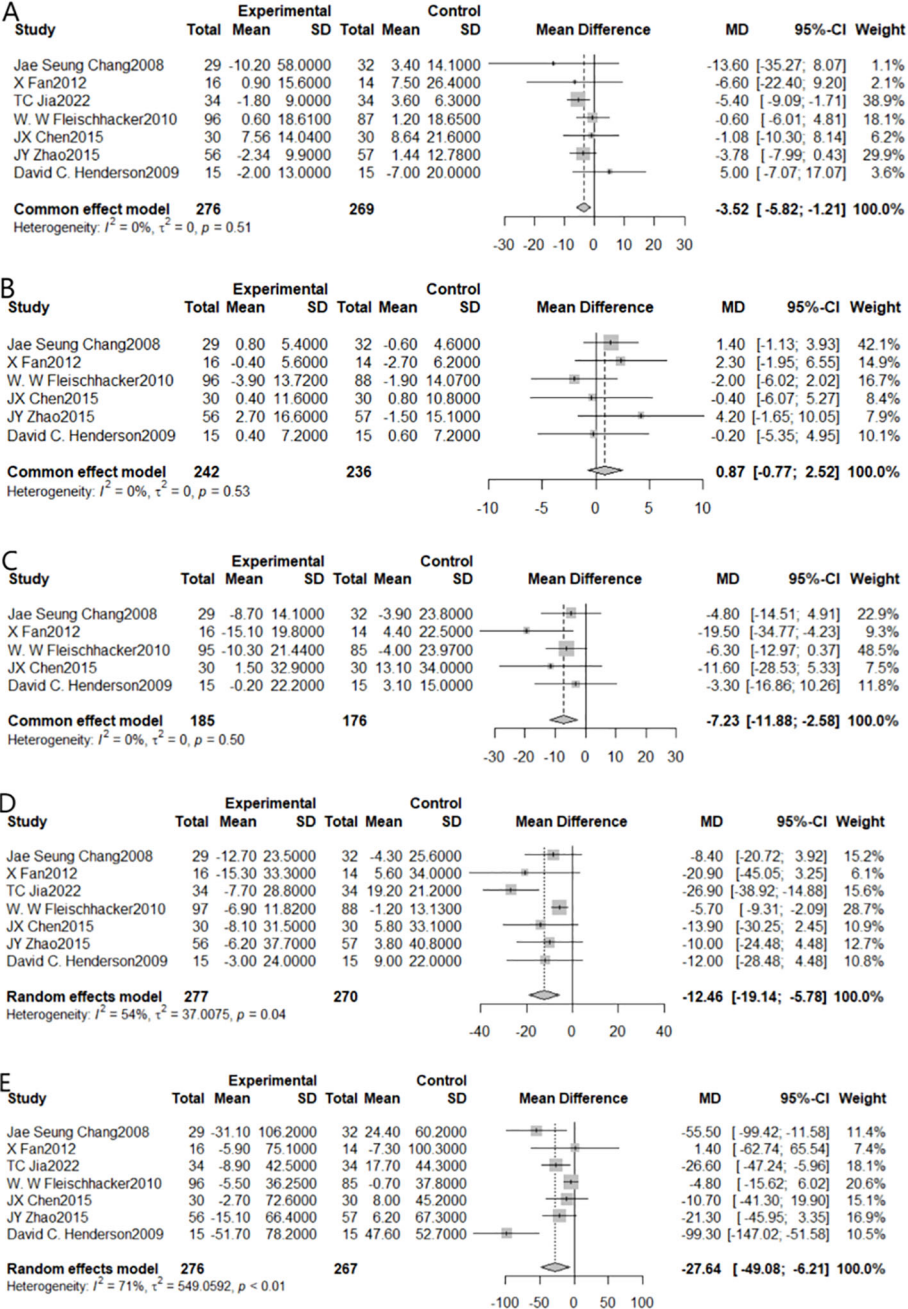
Forest plot of the results of all metabolic indicators in patients after combining aripiprazole [**(A)** for GLU, **(B)** for HDL, **(C)** for LDL, **(D)** for TC, **(E)** for TG].

The impact of aripiprazole adjunctive treatment on glycolipid metabolism in individuals with schizophrenia was evaluated using GLU as the outcome indicator, and seven studies satisfied the inclusion criteria (**Figure 3A**). Due to statistically insignificant heterogeneity (I^2^ = 0%, *P*=0.51), the fixed effect model was employed for the meta-analysis. Taking into account the sum of the effects, we found a statistically significant reduction [MD=-3.52, 95% CI (-5.82, -1.21)]. On the null line’s left side is a diamond-shaped square, which did not cross the line, indicating that adjuvant treatment with aripiprazole compared to the control group can effectively reduce blood glucose levels.

### 3.3 Effect on high-density lipoprotein levels

When HDL was chosen as the outcome indicator, 6 studies could be included (**Figure 3B**). Due to statistically insignificant heterogeneity (I^2^ = 0%, *P*=0.53), for the meta-analysis, we employed the fixed effect model. The results showed that the synergistic impact was not statistically significant [MD=0.87, 95% CI (-0.77,2.54)]. Adjuvant treatment with aripiprazole was not more effective than monotherapy in lowering HDL levels, as shown by the fact that the diamond-shaped squares and the null line crossed.

### 3.4 Effect on low-density lipoprotein levels

When LDL was chosen as the outcome indicator, 5 studies could be included (**Figure 3C**). Due to negligible heterogeneity, the meta-analysis used a fixed-effects model (I²=0%, *P*<0.01). The results showed a statistically significant combined effect [MD=-7.23, 95% CI (-11.88, -2.58)]. The square with the diamond form was positioned to the left of the null line, non-intersecting, indicating that adjuvant treatment with aripiprazole was effective in reducing LDL levels compared to the control group.

### 3.5 Effect on total cholesterol levels

When TC was chosen as the outcome indicator, 7 studies could be included (**Figure 3D**). Due to statistically significant heterogeneity (I^2^ = 54%, *P*=0.04), we performed Meta-analysis using a random effects model. The results showed a statistically significant combined effect [MD=-12.46, 95% CI (-19.14, -5.78)]. Since the diamond-shaped square did not cross the null line (just on the line’s left side), indicating that adjuvant treatment with aripiprazole was effective in reducing total cholesterol levels compared to the control group.

### 3.6 Effect on triglyceride levels

When TG was chosen as the outcome indicator, 7 studies could be included (**Figure 3E**). Due to statistically significant heterogeneity (I^2^ = 71%, *P* < 0.01), we used a random effects model for our meta-analysis. The results indicated the statistical significance of the summative impact [MD=-27.64, 95% CI (-49.08, -6.21)]. The square with the diamond form was positioned to the left of the zero-line, non-intersecting, indicating that adjuvant treatment with aripiprazole was effective in reducing triglyceride levels compared to the control group.

### 3.7 Effects on psychiatric symptoms

**Figure 4:**
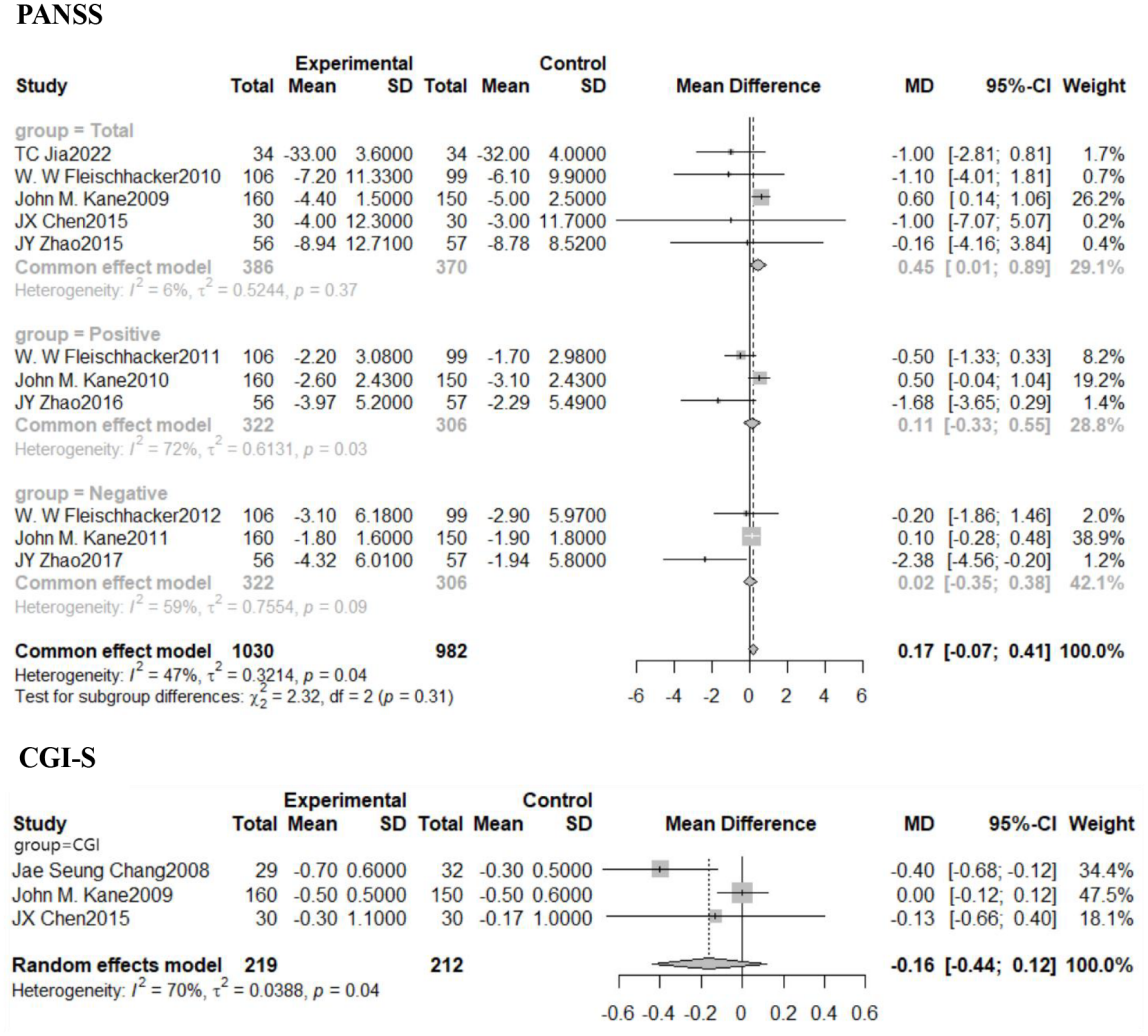
Forest plot of PANSS and CGI scores in patients after combination aripiprazole. PNASS, Positive and Negative Symptom Scale; CGI-S, Clinical Global Impressions-Severity of Illness Scale.

Statistically significant variations in PANSS scale total scores were seen between the treatment and placebo groups in psychiatric examinations using the PANSS and the CGI. Positive and negative PANSS scale ratings were not different between the treatment and placebo groups. Of these, a total of 5 studies pinned to the PANSS scale total score were included, and Meta-analysis was performed using a fixed-effects model due to statistically non-significant heterogeneity (I^2^ = 6%, *P*=0.37). There was a statistically significant combined impact [MD=0.45, 95% CI (0.01,0.89)], with the diamond-shaped square to the right of the null line, i.e., in favor of the placebo-treated side, and not intersecting the null line. This means that adjuvant treatment with aripiprazole leads to an increase in PANSS scores compared to control and a worsening of clinical symptoms compared to placebo. There was no statistically significant change in the CGI scale total score between the treatment and placebo groups, while the overall impact from the three trials using the CGI scale was not statistically significant [MD=-0.16, 95% CI (-0.44,0.12)]. Details are shown in **Figure 4**.

### 3.8 Effects on adverse reactions

Aripiprazole-assisted therapy did not significantly improve difficulties to sit still compared to placebo treatment (**Supplementary Figure S1**), along with back pain, gastrointestinal reactions, salivation, dizziness, dry mouth, insomnia, somnolence, and vomiting reactions.

Combination aripiprazole therapy was associated with a higher risk of nausea and vomiting than monotherapy, as evidenced by four RCTs reporting a statistically significant difference between the aripiprazole-adjuvant group and the control group (OR = 4.15, 95% CI: 1.93-8.91).

At least one extrapyramidal adverse event was documented in four RCTs. They discovered a statistically significant difference between the aripiprazole-adjuvant treatment group and the control group (OR = 1.83, 95% CI:1.18-2.84), with the combination treatment group having a greater risk of extrapyramidal adverse events than the monotherapy group.

In conclusion, combined aripiprazole therapy was generally well tolerated and safe, except for manifestations such as nausea and vomiting and extrapyramidal reactions.

### 3.9 Subgroup analyses, sensitivity analyses, and publication bias

We performed subgroup analyses of the main outcome indicators, including blood glucose, triglycerides, total cholesterol, HDL and LDL. The results of the subgroup analyses were shown in **Supplementary Figures S2**–**S6**. The effect of dose on metabolic indicators is often not negligible, but relevant studies are few and unclear (46). This is because the experimental group with combined aripiprazole treatment had a higher total dose, and it would be unfair not to increase the total dose in the control group (47). Therefore, we wanted to compare whether combined aripiprazole treatment with different doses would affect metabolic indices differently by subgroup analyses. In this study, the dose of combined aripiprazole therapy showed a significant difference after grouping the doses according to ≥15 mg and <15 mg, proving that aripiprazole greater than 15 mg improved the metabolic indexes more significantly.

A previous meta-analysis reported an effect of time of adjunctive aripiprazole therapy on outcome indicators (48), In our study the duration of treatment was grouped by ≤ 8 weeks and >8 weeks, and all metabolic indicators showed significant differences except for HDL. Metabolic improvement was more pronounced with combined treatment for ≤ 8 weeks than for >8 weeks, which showed significant differences except in terms of high-density lipoproteins, and metabolic improvement was more pronounced with combined treatment for ≤8 weeks than for >8 weeks, which may be due to the fact that long term use of aripiprazole tends to share the metabolic profile of other antipsychotic medications (49), and therefore combined aripiprazole should be used in short- to medium-term.

Meanwhile, metabolic indicators improved in the clozapine and olanzapine groups with aripiprazole-assisted medication, but there was no any significant improvement in the risperidone group (50). Aripiprazole may share the same metabolic profile as risperidone, which triggered a thought as to whether the difference in the type of the other drug may also have an effect on the effectiveness of aripiprazole-assisted therapy. Therefore, in this study, the included literatures were divided into clozapine, olanzapine and risperidone groups, indicating a statistically significant difference between the olanzapine and clozapine and risperidone groups. In addition, the metabolic improvement shown with combination aripiprazole treatment was more evident in the olanzapine group.

## 4 Discussion

The FDA has authorized the third-generation antipsychotic drug aripiprazole for the treatment of schizophrenia. In addition to its agonistic and antagonistic actions on certain D2 and 5-HT1A receptors, aripiprazole was available in both oral and long-acting injectable (LAI) stockpiling forms (51). As aripiprazole has a greater affinity for dopamine receptors (52) and has agonistic actions on D2 receptors (53), it may be reasonable to conduct the aforementioned combination experiments while some metabolic issues may be countered by partial agonist activity on 5-HT receptors (35). Most antipsychotics are clinically active when D2 receptor occupancy is about 65%, and when D2 receptor occupancy is over 80%, extrapyramidal side effects may occur; on the other hand, at 6-9mg/d, a peak is reached in dopamine D2 receptor accuracy (54), positron emission tomography (PET) studies have found that at a dose of 10mg/day, aripiprazole exhibits high occupancy rates at the D2 receptors in the striatum (caudate nucleus: 87%; putamen: 93%; ventral striatum: 91%), and the average occupancy rate at the 5-HT2 receptors is approximately 54%-60%, while the average occupancy rate at the 5-HT1A receptors is relatively low, around 16% (55). The pharmacological effects of aripiprazole on D2 receptors are unique, including not only partial agonism but also functionally selective effects on intracellular signaling pathways, which means that a D2 receptor occupancy rate of over 90% is required for aripiprazole to be clinically active, but relatively few side effects. Aripiprazole has bidirectional activity, serving as both an agonist and antagonist (postsynaptic) of D2 receptors, which can explain the fact that aripiprazole does not cause significant side effects despite high D2 receptor occupancy rates (56). Several long-term, follow-up studies have confirmed that aripiprazole is negative or non-significant for body weight when used as monotherapy, but may be protective for lipid metabolism (57, 58).

A previous meta-analysis found that switching to antipsychotic medications with lower metabolic risks or using combination therapies can improve lipid parameters in patients with schizophrenia. Nevertheless, it should be highlighted that meta-analysis focused on switching to drugs with reduced risk of metabolism, and as mentioned earlier, this strategy carries the risk of worsening clinical symptoms in some patients. Additionally, the data extraction in that meta-analysis had flaws, and the conclusion drawn by the authors that aripiprazole is superior to other lipid-lowering medications was overly hasty (59). In a comprehensive meta-analysis of 55 RCTs (n=4457) (36), it was found that combined aripiprazole significantly improved clinical symptoms, but did not involve data related to glycolipid metabolism. This finding contradicts the conclusion of our study that combined aripiprazole worsens clinical symptoms. This discrepancy may be due to the fact that this review primarily focused on metabolic parameters as outcome indicators and had insufficient inclusion of studies related to clinical symptoms. Many of the excluded studies only examined clinical symptoms without considering metabolic parameters. Therefore, the conclusions of this review regarding clinical symptoms may be inaccurate. However, a meta-analysis (Henderson Ferker Foundation 2013) explained the pharmacological strategies of antipsychotic drugs on weight gain and metabolic adverse effects, which also supported our research findings (60).

Currently, some clinical guidelines have now changed from their previous view of monotherapy. The National Institute for Health and Care Excellence (NICE) guideline “Adult psychosis and schizophrenia: Prevention and management,” issued in 2014 and reviewed five years after, argues against using combination of antipsychotics on a daily basis unless it is for a temporary measure like drug switching. However, if clozapine monotherapy proves to be ineffective, the guideline allows for the addition of additional antipsychotics to augment the therapeutic effect of the patient. Both the study by Pilinger et al. (61). and our study emphasize that monotherapy does not show significant clinical improvement for positive symptoms; antipsychotics exhibit a restricted effectiveness in enhancing adverse symptoms among schizophrenia patients (30); by using a combination of antipsychotics, it may allow a decrease in the dosage of another drug (62), save medication expenses, lessen the severity of extrapyramidal side effects (26). This development is encouraging, and as more researches are reported, combination therapy is becoming more widely accepted. More consideration should be given to the possibilities of combination medication in the development of guidelines, and biases against combination therapy should be discarded to better serve clinical practice.

In addition, our study is subject to other shortcomings. The lack of comprehensive coverage of metabolic parameters in the included studies may result in an inadequate representation of the data, which could potentially impact the accuracy of the results. The brief follow-up period of the included study precludes insight into the long-term safety and efficacy of the treatment regimen. The lack of diversity in age, gender, and comorbidities among the included patients prevented an adequate validation of differences in the impact of metabolic parameters of aripiprazole in different populations. It is recommended that subsequent studies should assess the frequency and severity of adverse effects in adjuvant therapy with aripiprazole, in order to provide clinicians with clearer guidance on risks.
