# Supplementary material for: A systematic review and meta-analysis of the effects of combined aripiprazole on glycolipid metabolism in schizophrenia
Source: Front Psychiatry. 2025 Jan 10;15:1496986. doi: 10.3389/fpsyt.2024.1496986 (PMC11757238; doi:10.3389/fpsyt.2024.1496986)
Supplement: Supplementary file 1 [file DataSheet1.pdf]

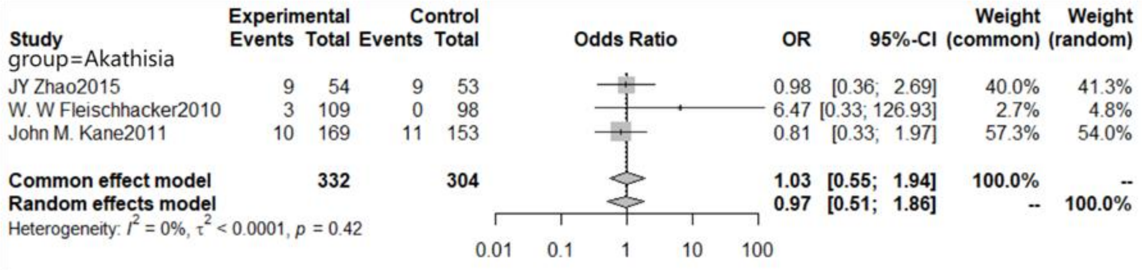
Experimental Control
Study Events Total Events Total Odds Ratio OR 95%-CI Weight (common) Weight (random)
group=Akathisia
JY Zhao2015 9 54 9 53 0.98 [0.36; 2.69] 40.0% 41.3%
W. W Fleischhacker2010 3 109 0 98 6.47 [0.33; 126.93] 2.7% 4.8%
John M. Kane2011 10 169 11 153 0.81 [0.33; 1.97] 57.3% 54.0%
Common effect model 332 304 1.03 [0.55; 1.94] 100.0% --
Random effects model 0.97 [0.51; 1.86] -- 100.0%
Heterogeneity: I^2 = 0%, τ^2 < 0.0001, p = 0.42
0.01 0.1 1 10 100

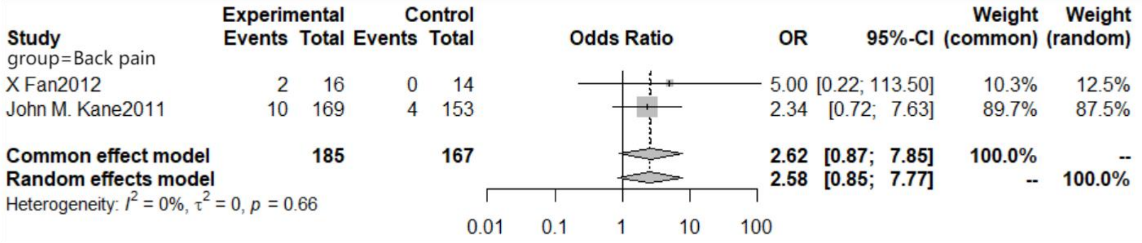
Experimental Control
Study Events Total Events Total Odds Ratio OR 95%-CI Weight (common) Weight (random)
group=Back pain
X Fan2012 2 16 0 14 5.00 [0.22; 113.50] 10.3% 12.5%
John M. Kane2011 10 169 4 153 2.34 [0.72; 7.63] 89.7% 87.5%
Common effect model 185 167 2.62 [0.87; 7.85] 100.0% --
Random effects model 2.58 [0.85; 7.77] -- 100.0%
Heterogeneity: I^2 = 0%, τ^2 = 0, p = 0.66
0.01 0.1 1 10 100

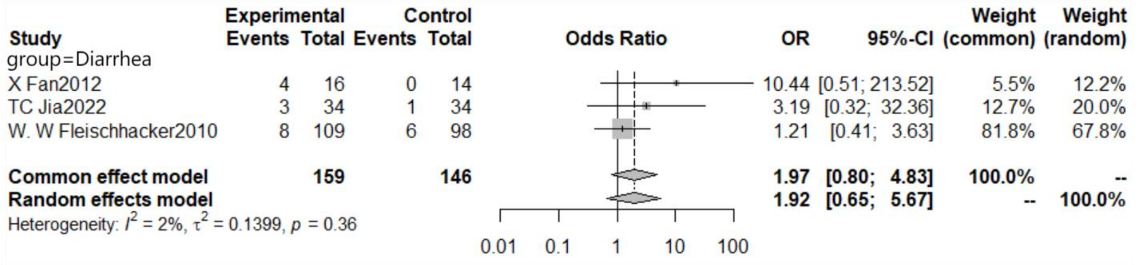
Experimental Control
Study Events Total Events Total Odds Ratio OR 95%-CI Weight (common) Weight (random)
group=Diarrhea
X Fan2012 4 16 0 14 10.44 [0.51; 213.52] 5.5% 12.2%
TC Jia2022 3 34 1 34 3.19 [0.32; 32.36] 12.7% 20.0%
W. W Fleischhacker2010 8 109 6 98 1.21 [0.41; 3.63] 81.8% 67.8%
Common effect model 159 146 1.97 [0.80; 4.83] 100.0% --
Random effects model 1.92 [0.65; 5.67] -- 100.0%
Heterogeneity: I^2 = 2%, τ^2 = 0.1399, p = 0.36
0.01 0.1 1 10 100

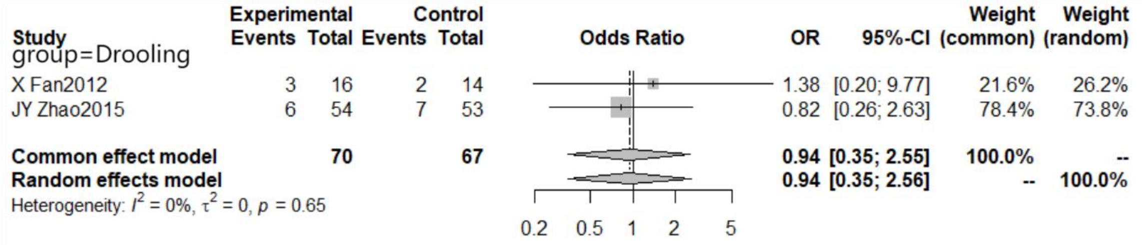
Experimental Control
Study Events Total Events Total Odds Ratio OR 95%-CI Weight (common) Weight (random)
group=Drooling
X Fan2012 3 16 2 14 1.38 [0.20; 9.77] 21.6% 26.2%
JY Zhao2015 6 54 7 53 0.82 [0.26; 2.63] 78.4% 73.8%
Common effect model 70 67 0.94 [0.35; 2.55] 100.0% --
Random effects model 0.94 [0.35; 2.56] -- 100.0%
Heterogeneity: I^2 = 0%, τ^2 = 0, p = 0.65
0.2 0.5 1 2 5

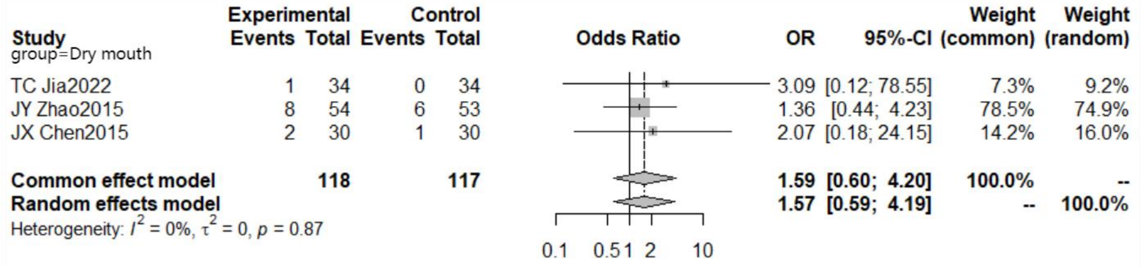
Experimental Control
Study Events Total Events Total Odds Ratio OR 95%-CI Weight (common) Weight (random)
group=Dry mouth
TC Jia2022 1 34 0 34 3.09 [0.12; 78.55] 7.3% 9.2%
JY Zhao2015 8 54 6 53 1.36 [0.44; 4.23] 78.5% 74.9%
JX Chen2015 2 30 1 30 2.07 [0.18; 24.15] 14.2% 16.0%
Common effect model 118 117 1.59 [0.60; 4.20] 100.0% --
Random effects model 1.57 [0.59; 4.19] -- 100.0%
Heterogeneity: I^2 = 0%, τ^2 = 0, p = 0.87
0.1 0.51 2 10

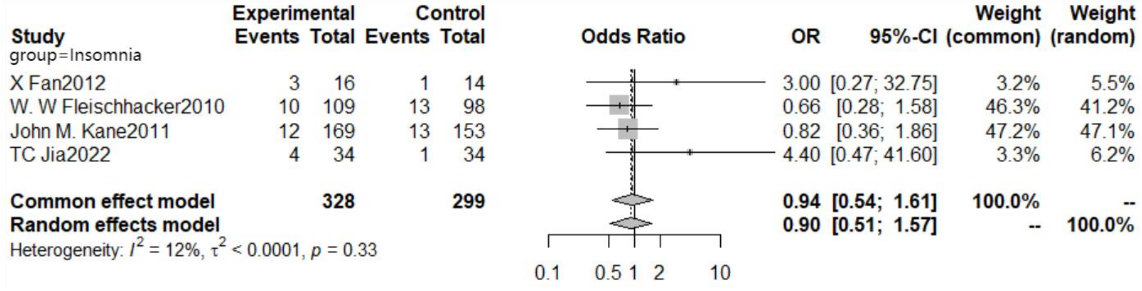
Experimental Control
Study Events Total Events Total Odds Ratio OR 95%-CI Weight (common) Weight (random)
group=Insomnia
X Fan2012 3 16 1 14 3.00 [0.27; 32.75] 3.2% 5.5%
W. W Fleischhacker2010 10 109 13 98 0.66 [0.28; 1.58] 46.3% 41.2%
John M. Kane2011 12 169 13 153 0.82 [0.36; 1.86] 47.2% 47.1%
TC Jia2022 4 34 1 34 4.40 [0.47; 41.60] 3.3% 6.2%
Common effect model 328 299 0.94 [0.54; 1.61] 100.0% --
Random effects model 0.90 [0.51; 1.57] -- 100.0%
Heterogeneity: I^2 = 12%, τ^2 < 0.0001, p = 0.33
0.1 0.5 1 2 10

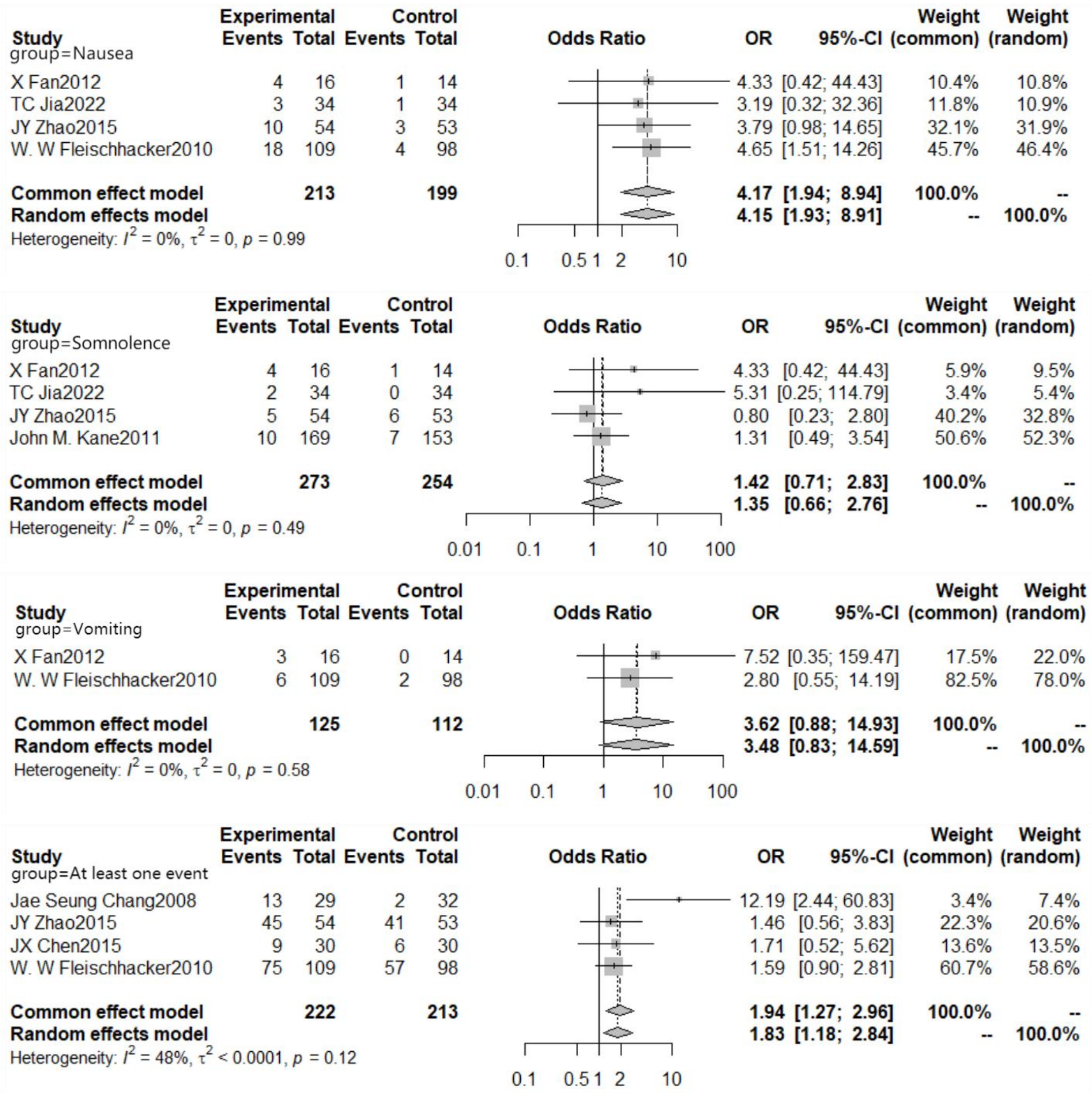

Figure S1 Forest plot of the risk of adverse reactions in patients after combining aripiprazole

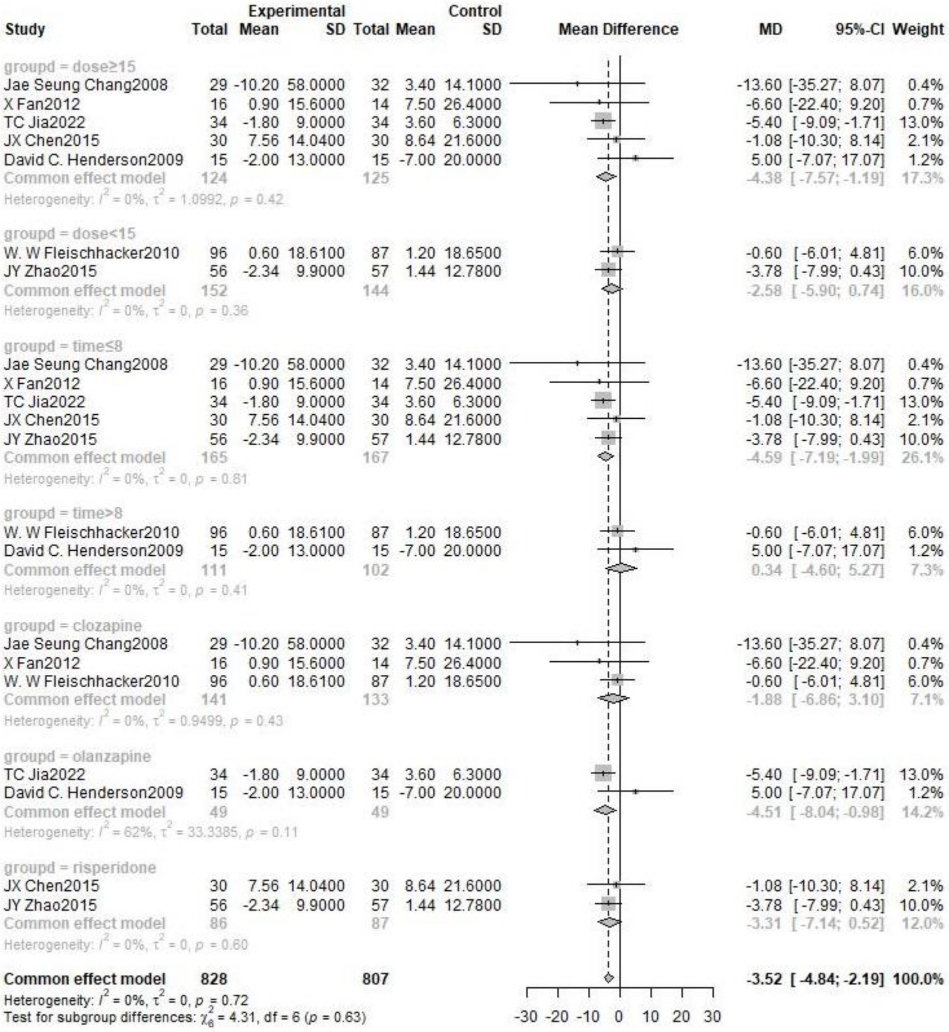

Figure S2 Results of subgroup analyses for blood glucose

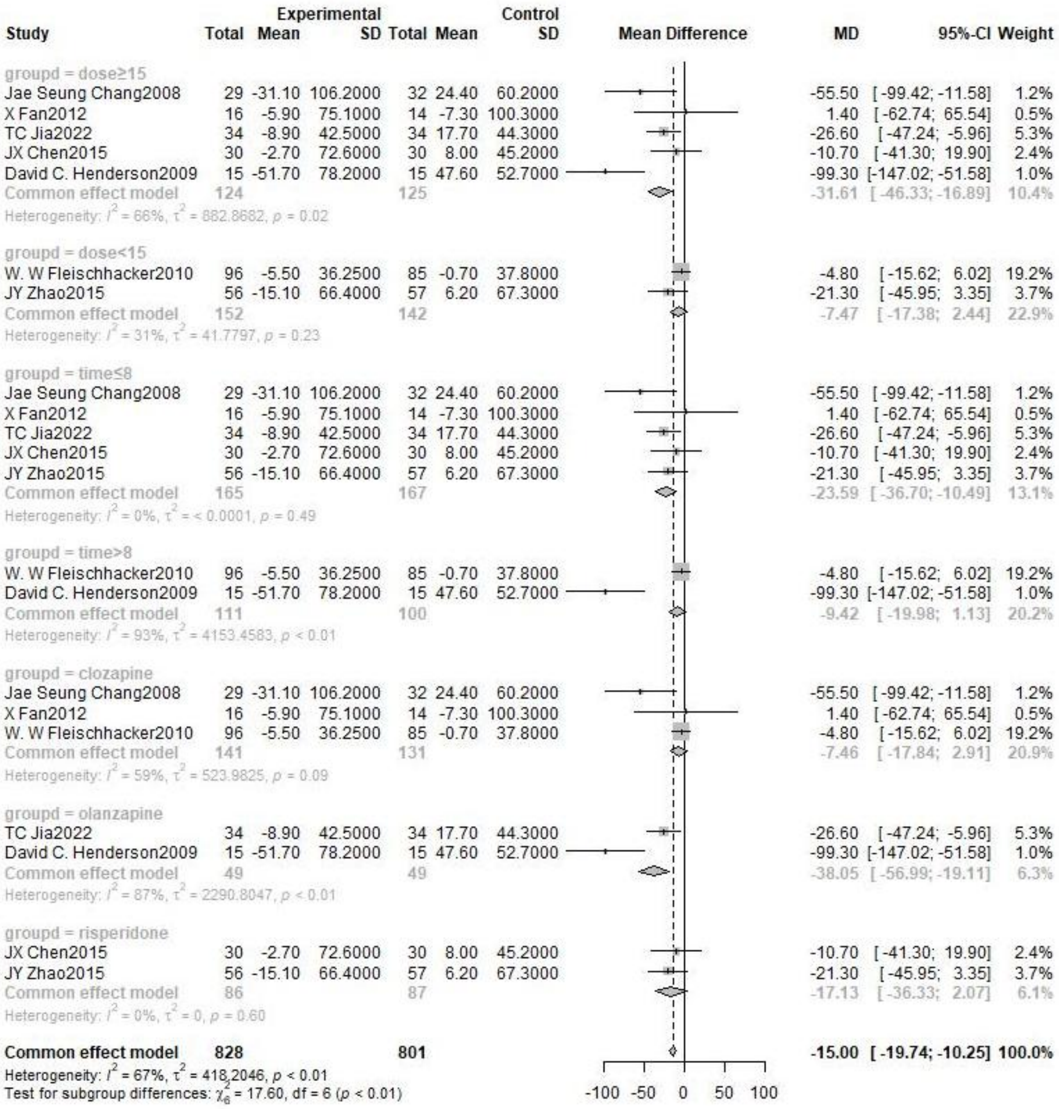

Figure S3 Results of subgroup analyses for triglyceride

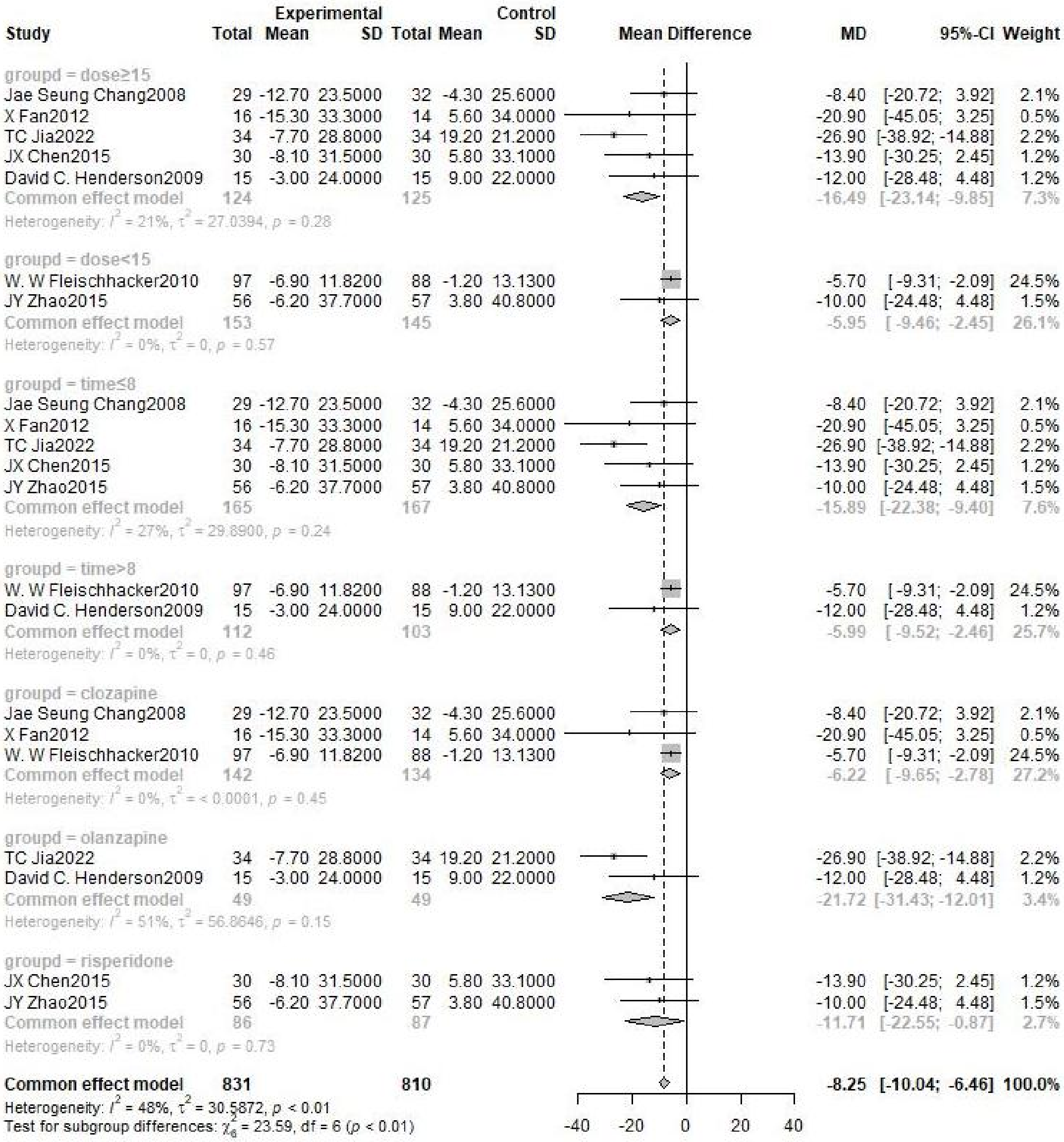

Figure S4 Results of subgroup analyses for total cholesterol

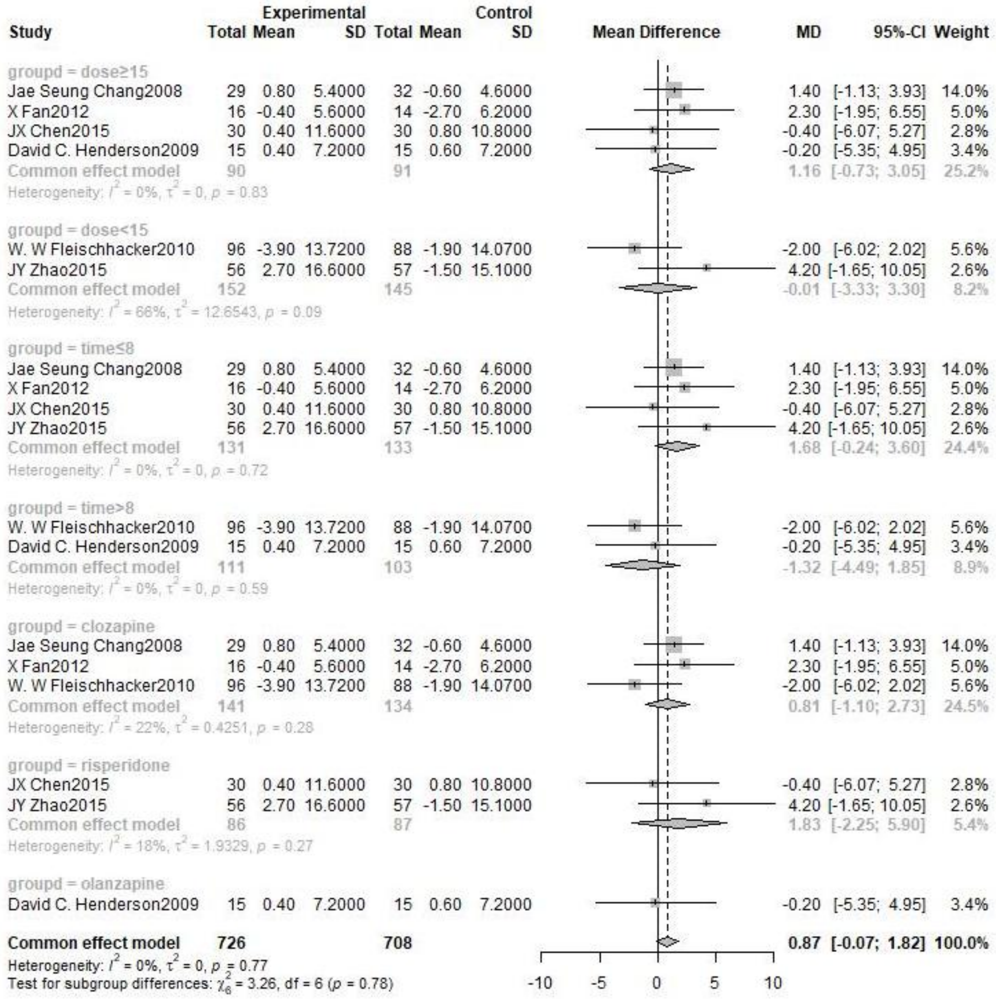

Figure S5 Results of subgroup analyses for high density lipoprotein

Study	Experimental Total	Experimental Mean	Experimental SD	Control Total	Control Mean	Control SD	MD	95%-CI	Weight
groupd = dose≥15									
Jae Seung Chang2008	29	-8.70	14.1000	32	-3.90	23.8000	-4.80	[-14.51; 4.91]	7.6%
X Fan2012	16	-15.10	19.8000	14	4.40	22.5000	-19.50	[-34.77; -4.23]	3.1%
JX Chen2015	30	1.50	32.9000	30	13.10	34.0000	-11.60	[-28.53; 5.33]	2.5%
David C. Henderson2009	15	-0.20	22.2000	15	3.10	15.0000	-3.30	[-16.86; 10.26]	3.9%
Common effect model	90			91			-8.10	[-14.58; -1.62]	17.2%
Heterogeneity: $I^2 = 7\%$, $\tau^2 = 1.6246$, $p = 0.36$									
groupd = dose<15									
W. W Fleischhacker2010	95	-10.30	21.4400	85	-4.00	23.9700	-6.30	[-12.97; 0.37]	16.2%
groupd = time≤8									
Jae Seung Chang2008	29	-8.70	14.1000	32	-3.90	23.8000	-4.80	[-14.51; 4.91]	7.6%
X Fan2012	16	-15.10	19.8000	14	4.40	22.5000	-19.50	[-34.77; -4.23]	3.1%
JX Chen2015	30	1.50	32.9000	30	13.10	34.0000	-11.60	[-28.53; 5.33]	2.5%
Common effect model	75			76			-9.52	[-16.90; -2.15]	13.2%
Heterogeneity: $I^2 = 23\%$, $\tau^2 = 20.3818$, $p = 0.27$									
groupd = time>8									
W. W Fleischhacker2010	95	-10.30	21.4400	85	-4.00	23.9700	-6.30	[-12.97; 0.37]	16.2%
David C. Henderson2009	15	-0.20	22.2000	15	3.10	15.0000	-3.30	[-16.86; 10.26]	3.9%
Common effect model	110			100			-5.71	[-11.70; 0.27]	20.1%
Heterogeneity: $I^2 = 0\%$, $\tau^2 = 0$, $p = 0.70$									
groupd = clozapine									
Jae Seung Chang2008	29	-8.70	14.1000	32	-3.90	23.8000	-4.80	[-14.51; 4.91]	7.6%
X Fan2012	16	-15.10	19.8000	14	4.40	22.5000	-19.50	[-34.77; -4.23]	3.1%
W. W Fleischhacker2010	95	-10.30	21.4400	85	-4.00	23.9700	-6.30	[-12.97; 0.37]	16.2%
Common effect model	140			131			-7.39	[-12.57; -2.22]	26.9%
Heterogeneity: $I^2 = 28\%$, $\tau^2 < 0.0001$, $p = 0.25$									
groupd = risperidone									
JX Chen2015	30	1.50	32.9000	30	13.10	34.0000	-11.60	[-28.53; 5.33]	2.5%
groupd = olanzapine									
David C. Henderson2009	15	-0.20	22.2000	15	3.10	15.0000	-3.30	[-16.86; 10.26]	3.9%
**Common effect model**	**555**			**528**			**-7.23**	**[-9.91; -4.54]**	**100.0%**
Heterogeneity: $I^2 = 0\%$, $\tau^2 = 0$, $p = 0.75$									
Test for subgroup differences: $\chi^2_6 = 1.34$, df = 6 ($p = 0.97$)									

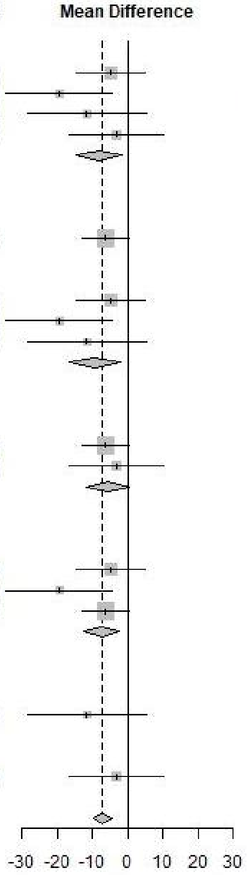

Figure S6 Results of subgroup analyses for low density lipoprotein
